# Supplementary material for: Baseline Characterization of the Gut Microbiota of Field and Colony Populations of Phlebotomus tobbi and Preliminary Assessment of the Anti-Leishmanial Activity of Cultivable Bacteria
Source: Pathogens. 2026 Jun 23;15(7):658. doi: 10.3390/pathogens15070658 (PMC13416292; doi:10.3390/pathogens15070658)
Supplement: Supplementary file 1 [file pathogens-15-00658-s001.zip › pathogens-4357168-File S1/Taxa2ASV_Decomposer/Genus/g__Sphingomonas/heatmap_with_clustering.pdf]

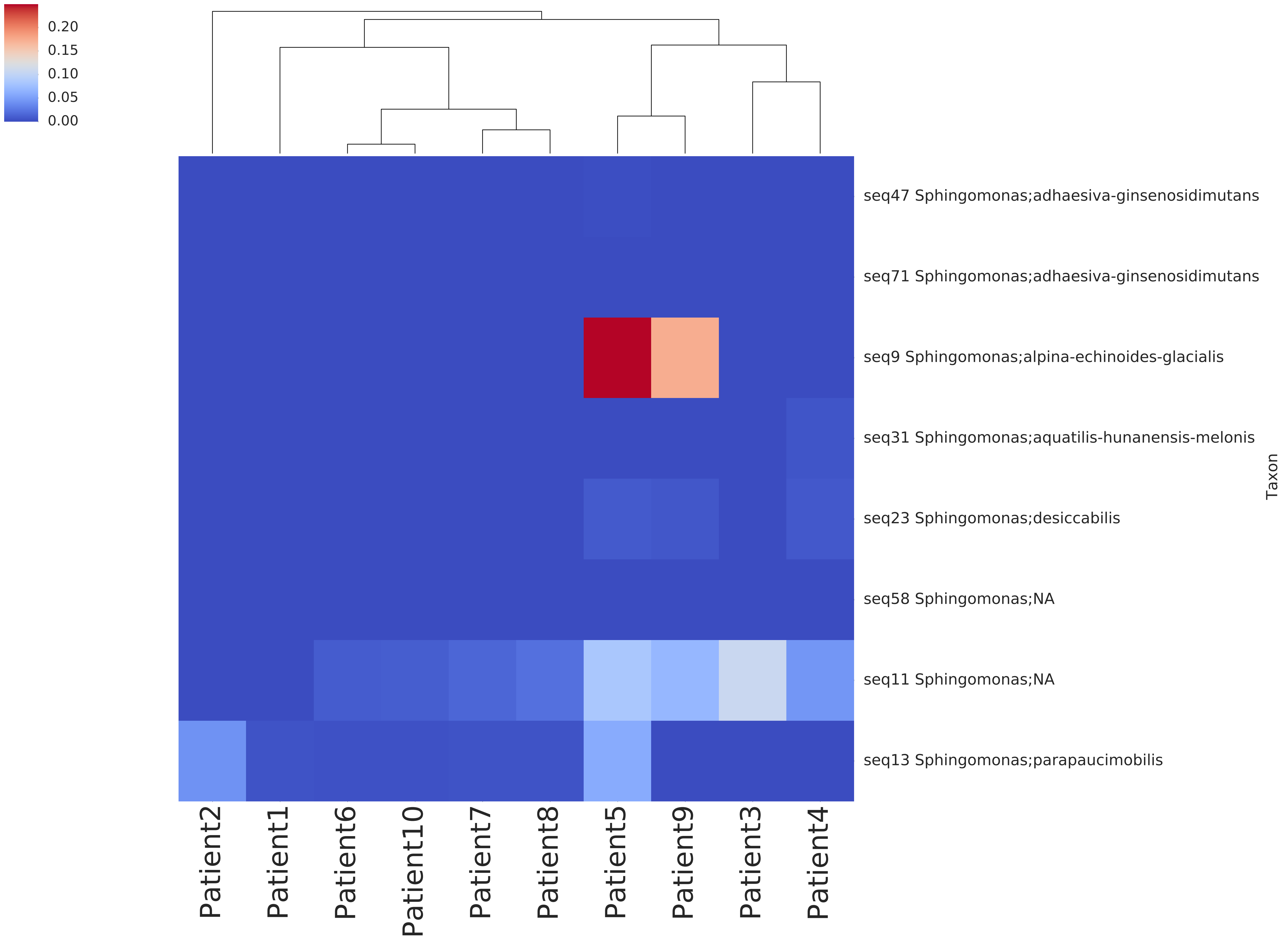
0.20
0.15
0.10
0.05
0.00
seq47 Sphingomonas;adhaesiva-ginsenosidimutans
seq71 Sphingomonas;adhaesiva-ginsenosidimutans
seq9 Sphingomonas;alpina-echinoides-glacialis
seq31 Sphingomonas;aquatilis-hunanensis-melonis
seq23 Sphingomonas;desiccabilis
seq58 Sphingomonas;NA
seq11 Sphingomonas;NA
seq13 Sphingomonas;parapaucimobilis
Taxon
Patient2
Patient1
Patient6
Patient10
Patient7
Patient8
Patient5
Patient9
Patient3
Patient4
